# Space‐Time Metasurface Antenna Enabled High‐Dimensional Electromagnetic Modulation for Intrinsic Physical Layer Security

**DOI:** 10.1002/advs.76676

**Published:** 2026-07-20

**Authors:** Xinyu Fang, Yiqing Sun, Chenfeng Yang, Siran Wang, Zheng Xing Wang, Kaixu Wang, Geng‐Bo Wu

**Affiliations:** ^1^ State Key Laboratory of Terahertz and Millimeter Waves and Department of Electrical Engineering City University of Hong Kong Hong Kong SAR China; ^2^ School of Electronics and Information Engineering Harbin Institute of Technology Shenzhen China

**Keywords:** electromagnetic wave, metasurface, secure communication, space‐time modulation

## Abstract

Physical‐layer security (PLS) plays a critical role in modern wireless communication systems by exploiting the fundamental properties of the physical medium to safeguard data transmission. Classical spread‐spectrum techniques enhance PLS through frequency‐bandwidth expansion, yet they typically exploit only a single electromagnetic (EM) degree of freedom for information mapping. Moreover, the separation between information mapping and spectrum spreading processes further constrains their spectral efficiency, system flexibility, and intrinsic security. Here, we introduce a high‐dimensional EM modulation paradigm for PLS communications enabled by a compact 1‐bit waveguide‐integrated space‐time metasurface antenna (STMA). By combining direct modulation of the +1 harmonic with controllable phase, frequency, and pseudorandom modulation with adjustable time‐delay, the STMA functions as an active information‐processing platform that embeds information directly into the physical radiation process. In contrast to conventional spread‐spectrum techniques, security enhancement in this design arises from expansion of the effective EM state space rather than bandwidth inflation alone, leading to improved spectral efficiency and resilience against both jamming and eavesdropping. These findings establish EM dimensionality engineering as a viable pathway toward secure and spectrally efficient wireless systems.

## Graphical abstract

The concept of high‐dimensional EM modulation using the STMA for physical layer secure communication is illustrated in figure. The STMA consists of a series of programmable unit cells, with 1‐bit reconfigurable phase states (0/π). The STMA is excited by a monochromatic continuous wave (CW) at frequency *f_c_* and controlled by a field‐programmable gate array (FPGA) platform. Through coordinated space‐time modulation, the radiated wave intrinsically carries information during the physical radiation process across three parallel EM dimensions, i.e., frequency, phase, and delay. Information bits mapped within these dimensions are represented by distinct color markers, highlighting their orthogonal or weakly correlated nature. Reliable demodulation requires prior knowledge of the multidimensional mapping manifold. Therefore, legitimate receivers positioned within the designated radiation direction can effectively reconstruct the transmitted information with high fidelity. In contrast, unauthorized observers, i.e., eavesdroppers, located outside the intended spatial channel encounter reduced detectability and pronounced dimensional mismatch, which substantially impairs signal recovery.

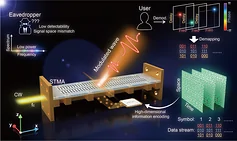

## 1 Introduction

The rapid proliferation of Internet of Things (IoT) devices and the transition toward 6G wireless networks are redefining the paradigm of global connectivity [1, 2]. Future wireless infrastructures are expected to simultaneously support massive device density, ultra‐low latency, and ubiquitous access. Yet the broadcast nature of electromagnetic (EM) wave propagation inherently exposes wireless transmission to interception, spoofing, and intentional jamming. As connectivity scales, so too does the attack surface. Ensuring information security is therefore not merely desirable, but foundational for next‐generation wireless ecosystems. Conventional security frameworks predominantly rely on application‐layer cryptography [3], whose robustness is rooted in computational complexity. While highly effective in many scenarios, such approaches inevitably introduce processing overhead and key‐management burdens and face increasing vulnerability in the era of quantum computing and resource‐constrained environments. Physical‐layer security (PLS) offers an alternative paradigm by leveraging the inherently stochastic and structural properties of EM propagation channels [4]. Rather than relying on algorithmic hardness, PLS seeks to embed security into the physics of signal generation and transmission.

Among various PLS techniques, spread‐spectrum communication has long been recognized as a particularly effective strategy owing to its inherent resistance to interference and low probability of detection (LPD) [5, 6]. By distributing signal energy across a wider bandwidth using pseudorandom codes, spread‐spectrum systems obscure the transmitted information and improve robustness against interference and jamming. However, its security enhancement primarily arises from bandwidth expansion and energy dispersion. The fundamental dimensionality of the EM mapping space remains largely unchanged. As adversaries acquire wideband receivers, multi‐antenna arrays, and advanced signal processing capabilities, such bandwidth‐based obfuscation becomes increasingly insufficient. A deeper question thus emerges as to whether security can be enhanced by expanding the intrinsic EM state space itself, rather than merely redistributing spectral resources.

Recent advances in artificial EM materials [7, 8], particularly metasurfaces, have introduced unprecedented control over wavefront engineering. Early studies primarily focused on static metasurfaces that manipulate the spatial distribution of scattered fields [9, 10, 11], followed by reconfigurable metasurfaces [12] capable of dynamically altering phase or amplitude responses through the incorporation of tunable elements such as PIN diodes [13, 14], varactors [15, 16], MEMS [17, 18], liquid crystals [19], and graphene [20], etc. More recently, space‐time‐modulated metasurfaces further established dynamic coupling between spatial and temporal domains [21, 22]. These developments have unlocked novel functionalities, including harmonic beam steering [22, 23, 24, 25], Doppler cloaking [26, 27], wireless communications [28, 29, 30, 31, 32, 33, 34, 35, 36], radar deception [37, 38, 39, 40, 41], and direction of arrival (DoA) estimation [42, 43, 44, 45, 46, 47].

Despite these advances, most reported metasurface‐enabled communication schemes map information within a single modulation dimension, such as complex amplitude [28, 30, 31, 32, 34, 36], frequency shift [29, 33], or coding states [35], thereby limiting the effective signal manifold. Although pseudorandom modulation can broaden the signal bandwidth and enhance transmission security [31, 34, 35], it primarily redistributes spectral resources rather than expanding the EM state space available for information mapping. As a result, information mapping and secure modulation remain loosely coupled, constraining both the scalability of communication security and the achievable spectral efficiency.

In this article, we introduce a high‐dimensional EM modulation paradigm enabled by a space‐time metasurface antenna (STMA). By jointly employing direct modulation of the +1 harmonic with independently controllable phase and frequency, together with pseudorandom modulation incorporating programmable time‐delay, information is embedded across multiple EM degrees of freedom: phase, frequency, and delay. This multidimensional mapping intrinsically integrates information into the physical radiation process, transforming the STMA into an active information‐processing entity. The resulting signaling mechanism simultaneously achieves spatially directional transmission, agile frequency‐delay varying, and LPD. Legitimate receivers, equipped with prior knowledge of the modulation manifold, can reliably recover the transmitted information, whereas unauthorized observers encounter severe dimensional mismatch and reduced detectability.

Security enhancement in this framework arises from EM state space expansion rather than relying only on bandwidth inflation [4, 5, 6, 31, 34]. This dimensional gain enables improved spectral efficiency and resilience against both jamming and eavesdropping. Moreover, all these high‐dimensional modulation and security enhancement functionalities are consolidated within a compact waveguide‐integrated STMA employing just 1‐bit reconfigurable phase states, yielding a hardware‐efficient architecture. Experimental validation confirms robust high‐dimensional secure transmission, establishing EM dimensionality engineering as a viable route toward intrinsically secure and spectrally efficient wireless systems for future IoT and 6G networks.

## 2 Theory and Modeling

Figure 1 illustrates the concept of high‐dimensional EM modulation using the STMA for physical layer secure communication. The STMA consists of a series of programmable unit cells, with 1‐bit reconfigurable phase states (0/π). The STMA is excited by a monochromatic continuous wave (CW) at frequency *f_c_* and controlled by a field‐programmable gate array (FPGA) platform. Through coordinated space‐time modulation, the radiated wave intrinsically carries information during the physical radiation process across three parallel EM dimensions, i.e., frequency, phase, and delay. Information bits mapped within these dimensions are represented by distinct color markers, highlighting their orthogonal or weakly correlated nature. Reliable demodulation requires prior knowledge of the multidimensional mapping manifold. Therefore, legitimate receivers positioned within the designated radiation direction can effectively reconstruct the transmitted information with high fidelity. In contrast, unauthorized observers, i.e., eavesdroppers, located outside the intended spatial channel encounter reduced detectability and pronounced dimensional mismatch, which substantially impairs signal recovery.

**FIGURE 1:**
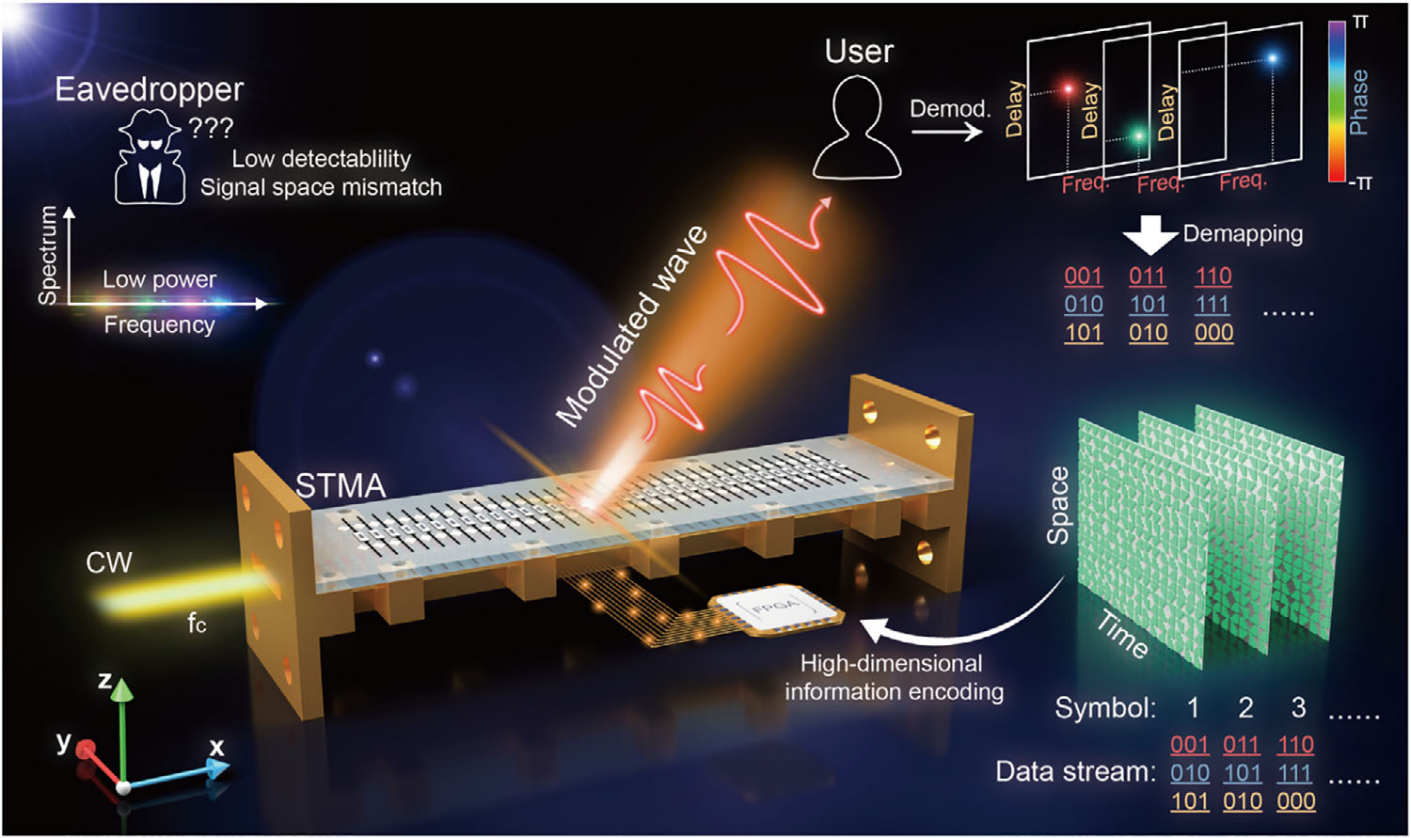
Conceptual illustration of the high‐dimensional EM modulation using the STMA for physical layer security. The STMA, composed of reconfigurable unit cells, is powered by a monochromatic CW at frequency *f_c_* and driven by an FPGA platform for space‐time modulation. Baseband information bits are mapped in frequency (red), phase (blue), and delay (yellow), respectively. CW, continuous wave; FPGA, field‐programmable gate array; STMA, space‐time metasurface antenna.

### 2.1 Far‐Field Radiation of High‐Dimensional Modulated STMA

We first start by analyzing the far‐field radiation characteristics of the proposed high‐dimensional modulated STMA. Assume that the waveguide‐integrated metasurface is excited by a monochromatic CW signal $e^{j2\pi f_{c}t}$ generated by a local oscillator (LO). The legitimate user and eavesdropper are located in the far‐field region of the STMA at angular directions of θ_*u*_ and θ_*e*_ relative to the *z*‐axis, respectively. Considering a one‐dimensional (1D) configuration, the STMA consists of *N* unit cells arranged along the *x*‐axis with a subwavelength periodicity of *d*. Each unit cell supports 1‐bit phase reconfiguration (0/π), enabling digital phase control across the metasurface aperture. The modulation process comprises two distinct components: *m*(*t*), which governs the frequency and phase modulation, and *c*(*t*), which represents the spreading sequence used for delay modulation. Based on these two modulation components, the time‐varying modulation signal of the *n* ^th^ unit cell during the *s* ^th^ symbol interval can be expressed as:

(1) $$U_{n,s}\left(t\right)=m_{n}\;\left(t-t_{s}\right)c\left(t-\tau_{s}\right)$$

The symbol period of U_*n*,*s*_(*t*) is *T_p_*, corresponding to a symbol modulation frequency of *f_p_* = 1/*T_p_* . The corresponding symbol rate is therefore *f_p_* sym/s. *m_n_* (t) represents the direct modulation for the +1 harmonic beam of the *n* ^th^ unit cell and is defined as $m\left(t\right)=\left\{\begin{array}{cc} 1, & 0\leq t<T_{s}/2\\ -1, & T_{s}/2\leq t<T_{s} \end{array}\right.$, where *T_s_* is the modulation period of *m*(*t*) during the *s* ^th^ symbol interval, with $T_{s}=\frac{T_{p}}{k}\;,\;k=1,2,\text{…},K$. For frequency control of the radiated +1 harmonic, the modulation period *T_s_* determines its +1 harmonic modulation frequency, i.e., *f_s_* =  *kf_p_*. Without loss of generality, eight frequencies (*K* = 8) are adopted in this work for information mapping, as shown in Figure 2a.

**FIGURE 2:**
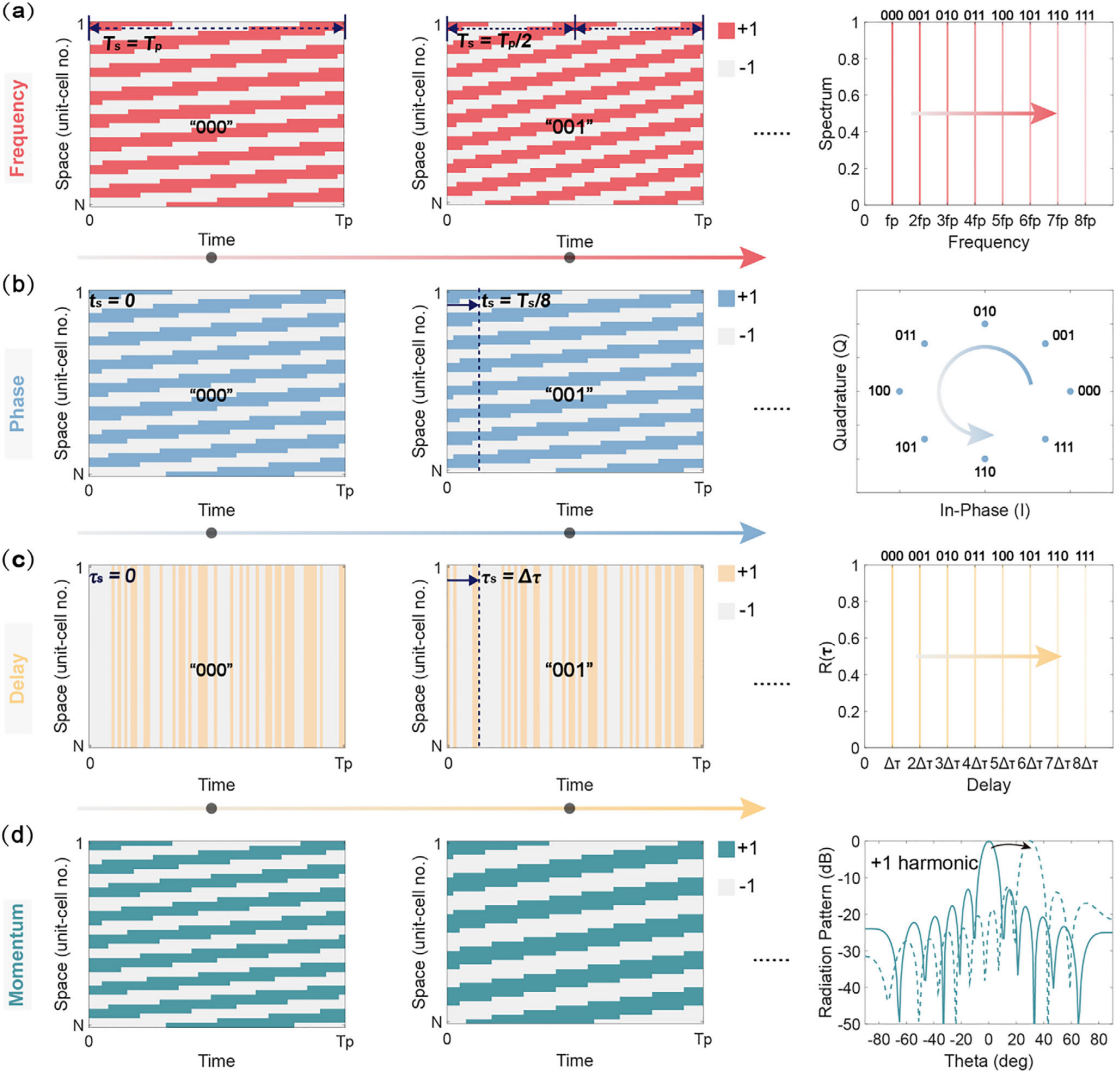
STMA high‐dimensional modulation scheme. (a) Direct modulation of the +1 harmonic with controllable frequency. (b) Direct modulation of the +1 harmonic with controllable phase. (c) Pseudorandom modulation incorporating programmable time‐delay. (d) Steering the +1 harmonic beam.

For phase control, a consistent time‐delay *t_s_* is applied across all unit cells, introducing a phase shift for the +1 harmonic such that $m_{n}\left(t-t_{s}\right)\overset{FS}{↔}\alpha_{n}^{h}e^{-j2\pi hf_{s}t_{s}}{|}_{h=+1}$, wherein $\alpha_{n}^{h}=\left\langle m_{n}\left(t\right),e^{j2\pi hf_{s}t}\right\rangle$ denotes the complex Fourier‐series coefficient of the *h* ^th^ harmonic for the *n* ^th^ unit cell. M‐ary phase‐shift keying (MPSK) can be realized by selecting *t_s_* as $t_{s}=-\frac{uT_{s}}{M},\;u=0,1,2,\text{…},\left(M-1\right)$. Without loss of generality, eight‐phase‐shift keying (8‐PSK, *M* = 8) is adopted in this work for information mapping, resulting in $t_{s}\in \left\{0,-\frac{T_{s}}{8},\text{…},-\frac{7T_{s}}{8}\right\}$, as shown in Figure 2b. The direct signal modulation scheme is also applicable to other information mapping formats, such as quadrature amplitude modulation (QAM). Further details regarding the implementation of the proposed 16‐QAM scheme are provided in Note S1 (Figures S1 and S2).

For delay control, *c*(*t* − τ_*s*_) is introduced in Equation (1). It denotes the pseudorandom sequence with programmable delay τ_*s*_ used for spectrum spreading, expressed as $c\left(t\right)=\sum_{l=1}^{L}a_{l}p\left[t-\left(l-1\right)T_{p}/L\right]$, where *T_p_* is the symbol period and *p*(*t*) is a rectangular sub‐pulse with a duration of *T_p_*/*L*. *c*(*t*) contains *L* rectangular sub‐pulses within each modulation period, where each pulse is weighted by a binary coefficient $a_{l}\in \left\{1,-1\right\}$. The same pseudorandom sequence *c*(*t* − τ_*s*_) is applied across all unit cells. The resulting spread‐spectrum waveform exhibits an approximate spectral envelope of $\sqrt{\frac{L+1}{L^{2}}}\mathrm{sinc}\left(\frac{T_{p}}{L}f\right)$, with a maximum amplitude of $\sqrt{\frac{L+1}{L^{2}}}$, and a main‐lobe bandwidth of 2*L*/*T_p_*, thereby producing a low‐power spread‐spectrum that enhances LPD performance [5, 6, 37]. The time‐delay τ_*s*_, defined as $\tau_{s}=\frac{T_{p}}{q}\;,\;q=1,2,\text{…},Q,$ can be used for information mapping. Owing to the strong autocorrelation properties of the pseudorandom sequence, the value of τ_*s*_ can be determined from the correlation peak at which the two signals are aligned [5, 6], $R\left(\tau \right)=\int_{0}^{T_{p}}c\left(t-\tau \right)c\left(t-\tau_{s}\right)\;dt$. The autocorrelation performance of *c*(*t*) is detailed in Note S2 (Figure S3). Without loss of generality, eight delays (*Q* = 8) is adopted in this work for information mapping, resulting in $\tau_{s}\in \left\{\frac{T_{p}}{8},\frac{2T_{p}}{8}\text{…},T_{p}\right\}$, with a step of $\Delta \tau =\frac{T_{p}}{8}$, as illustrated in Figure 2c.

For beam direction control, an additional time‐delay gradient along the metasurface aperture is further introduced to steer the +1 harmonic beam. Assuming that the modulation rate of the time‐varying signal U_*n*,*s*_(*t*) is much lower than the carrier frequency *f_c_*, the transmitted signal radiated by the STMA can be expressed as:

(2) $$E_{s}\left(t,\theta \right)=e^{j2\pi f_{c}t}\;\sum_{n=1}^{N}U_{n,s}\left(t\right)e^{j\xi_{c}\left(n-1\right)d\sin\theta }e^{-j\xi_{wg}\left(n-1\right)d}$$

wherein ξ_*c*_, ξ_*wg*_ denote the vacuum and the waveguide wavenumbers at the frequency *f_c_*, respectively. For the designed STMA, a slow‐wave transmission line is assumed with ξ_*wg*_ = 1.15ξ_*c*_ , while conductor and dielectric losses are neglected. Thanks to the slow guided wave that propagates along the length of the waveguide and sequentially excites each unit cell, distinct initial excitation phases are established across the aperture. Consequently, the STMA architecture can suppress undesired harmonic components, even with only 1‐bit phase modulation [25]. A detailed explanation of the undesired harmonic suppression mechanism is provided in Note S3 (Figure S4). After removing the carrier component at *f_c_*, the transmitted signal corresponding to the *s* ^th^ symbol can be expanded into a Fourier series as:

(3) $$\begin{array}{ccc} E_{s}\left(t,\theta \right) & = & c\left(t-\tau_{s}\right)\sum_{h=-\infty }^{\infty }\sum_{n=1}^{N}\alpha_{n}^{h}e^{j2\pi hf_{s}t}e^{-j2\pi hf_{s}t_{s}}e^{j\xi_{c}\left(n-1\right)d\sin\theta }\\ & & e^{-j\xi_{wg}\left(n-1\right)d} \end{array}$$

where the far‐field of the *h* ^th^ harmonic can be expressed as $AF^{h}\left(\theta \right)=\sum_{n=1}^{N}\alpha_{n}^{h}e^{j\xi_{c}\left(n-1\right)d\sin\theta }\;e^{-j\xi_{wg}\left(n-1\right)d}$. In this work, the *h* = +1 harmonic is employed for signal transmission, yielding the desired beam pattern $AF^{+1}\left(\theta \right)=\sum_{n=1}^{N}\alpha_{n}^{+1}e^{j\xi_{c}\left(n-1\right)d\sin\theta }\;e^{-j\xi_{wg}\left(n-1\right)d}$. As shown in Figure 2d, by appropriately designing the time‐delay μ_*n*_, i.e., *m_n_* (*t*)  =  *m*(*t* − μ_*n*_), $\mu_{n}\in \left[0,T_{s}\right]$, at different unit cells, the +1 harmonic can be steered toward the desired direction θ_*u*_, i.e., momentum manipulation, as detailed in Note S4.

The high‐dimensional EM modulation U_*n*,*s*_(*t*) integrates direct information modulation of the +1 harmonic with independently controllable phase and frequency, together with pseudorandom modulation incorporating programmable time‐delay. By embedding information directly into the physical radiation process, the scheme transforms modulation from a spectral allocation strategy into a multidimensional EM state space engineering problem. As a result, the effective signal space is significantly expanded, yielding simultaneous enhancement of intrinsic security and spectral efficiency. The resulting transmission inherently combines spatial directivity, spread‐spectrum‐based LPD, and agile frequency‐delay varying across symbols. Therefore, unauthorized receivers lacking prior knowledge of the modulation manifold encounter severe dimensional ambiguity, rendering reliable parameter estimation and signal reconstruction substantially more difficult.

The spectral efficiency of the high‐dimensional modulation scales with the accessible EM state space and can be expressed as $\eta =\frac{log_{2}KMQ}{L}$ bit/s/Hz, where *K*, *M*, *Q* denote the numbers of frequency, phase, and delay states, respectively, and *L* represents the number of pseudorandom sub‐pulses. The calculation of spectral efficiency is detailed in Note S5. In contrast, conventional spread‐spectrum schemes typically exploit a single modulation dimension [5, 6, 31, 34] for information mapping. For example, in MPSK, the spectral efficiency is given by $\eta =\frac{log_{2}M}{L}$ bit/s/Hz. This multiplicative dimensional scaling illustrates that security and spectral efficiency improvements arise from EM state space expansion rather than relying simply on bandwidth inflation or power redistribution.

### 2.2 Secure Communication Against Eavesdropping and Jamming

As shown in Figure 3a, for the legitimate user located at the desired direction θ_*u*_, the received signal during the *s* ^th^ symbol interval can be expressed as:

(4) $$\begin{array}{ccc} E_{s}\left(t,\theta_{u}\right) & = & c\left(t-\tau_{s}\right)e^{-j2\pi f_{s}t_{s}}e^{j2\pi f_{s}t}AF^{+1}\left(\theta_{u}\right)\\ & & +c\left(t-\tau_{s}\right)\sum_{\begin{array}{c} h=-\infty \\ h\neq +1 \end{array}}^{\infty }e^{-j2\pi hf_{s}t_{s}}e^{j2\pi hf_{s}t}AF^{h}\left(\theta_{u}\right) \end{array}$$

**FIGURE 3:**
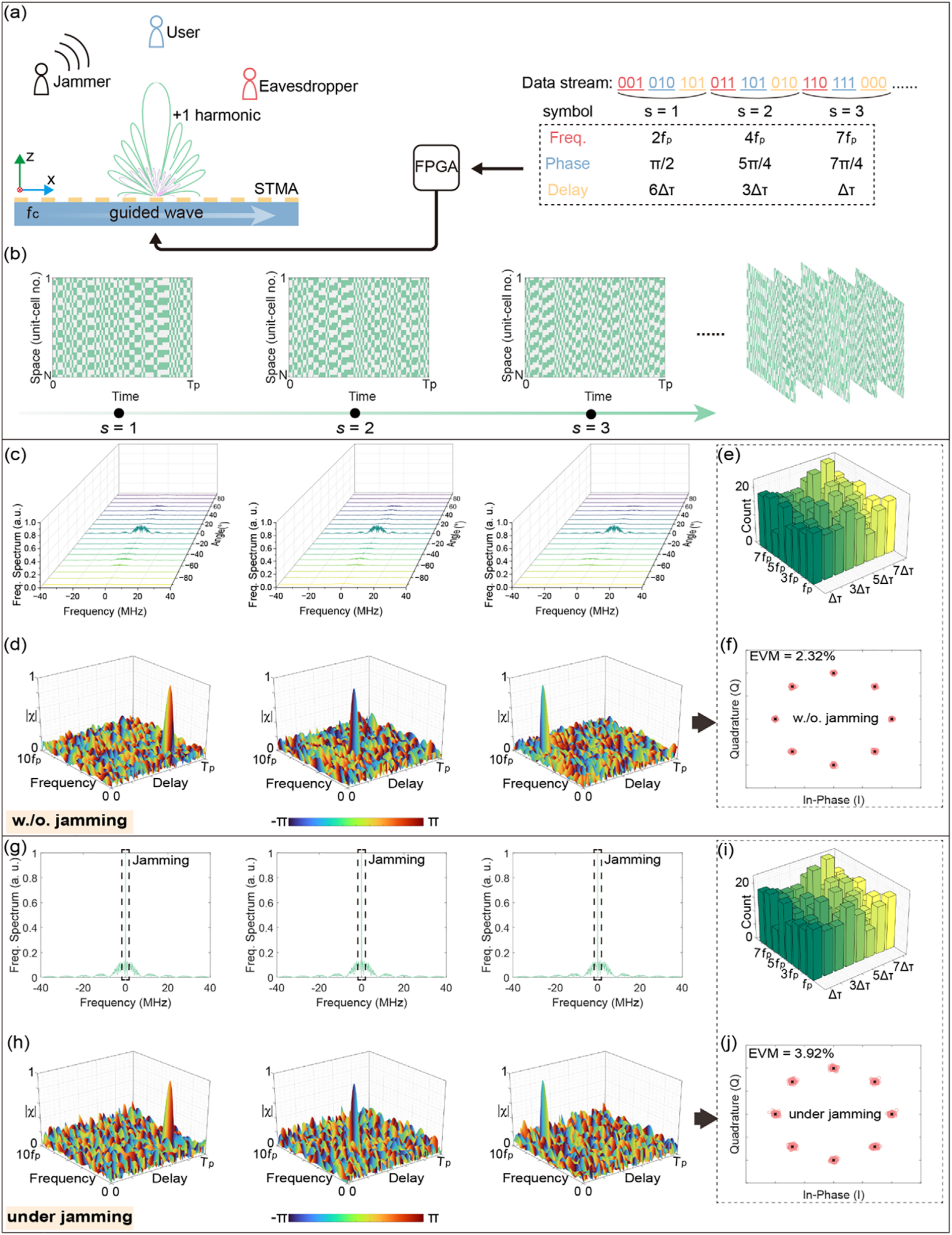
Simulated physical secure communication performance against eavesdropping and jamming. (a) Secure communication scenario with the user at 0° and an eavesdropper at 30°, in the presence of a jammer, illustrated with three transmitted symbols. (b) Space‐time modulation matrix U_*n*,*s*_(*t*) for symbols *s* = 1, 2, 3. (c) Received frequency spectra across all observation angles for *s* = 1, 2, 3, respectively. (d) 2D correlation detection results for the user for symbol *s* = 1, 2, 3, respectively. 1000 random symbols transmission (no jamming): (e) Statistical distribution of detected symbol frequencies and delays and (f) constellation diagram with EVM = 2.32%. (g) Frequency spectra under jamming of the user at θ_*u*_ =  0° for symbol *s* = 1, 2, 3, respectively. (h) 2D correlation results under jamming detected by the user for symbol *s* = 1, 2, 3, respectively. 1000 random symbols transmission (under jamming): (i) Statistical distribution of detected symbol frequencies and delays, and (j) constellation diagram with EVM = 3.92%.

The waveguide‐integrated structure of the STMA ensures that the +1 harmonic in the first term in Equation (4) dominates [25, 28], while the second term in Equation (4), corresponding to undesired sidebands, is a residual component with an amplitude much smaller than that of the first term and can therefore be neglected. As a result, the STMA produces a high‐power, unaliased, and spread‐spectrum signal in the desired direction. The legitimate user can recover the transmitted information using the pre‐shared pseudorandom sequence *c*(*t*) through two‐dimensional (2D) correlation detection [40]. The demodulated signal is expressed as:

(5) $$\chi_{s}\left(f,\tau \right)=\int_{\left(s-1\right)T_{p}}^{sT_{p}}E_{s}\left(t,\theta_{u}\right)\;c\left(t-\tau \right)e^{j2\pi ft}dt$$

When the frequency and delay match, i.e., *f*  = *f_s_* , τ  = τ_*s*_ , a peak appears in Equation (5) with a phase of φ  =   −2π*f_s_t_s_*. Therefore, Equation (5) effectively achieves joint detection across the three dimensions (phase, frequency, and delay). In contrast, an eavesdropper located in an undesired direction θ_*e*_ receives an attenuated +1 harmonic, resulting in a low‐power, distorted, and spread‐spectrum signal. Moreover, without prior knowledge of the pseudorandom sequence and modulation scheme, the unauthorized eavesdropper is unable to correctly recover the transmitted information due to severe signal distortion and dimensional mismatch, thereby achieving low detectability and effective eavesdropping protection.

Another advantage of the high‐dimensional modulation, particularly the pseudorandom component, is its inherent resilience to in‐band jamming. In the presence of jamming, the 2D correlation detection in Equation (5) can be expressed as:

(6) $$\begin{array}{ccc} \chi_{s}\left(f,\tau \right) & = & \int_{\left(s-1\right)T_{p}}^{sT_{p}}E_{s}\left(t,\theta_{u}\right)\;c\left(t-\tau \right)e^{j2\pi ft}dt\\ & & +\int_{\left(s-1\right)T_{p}}^{sT_{p}}J\left(t\right)c\left(t-\tau \right)e^{j2\pi ft}dt \end{array}$$

where *J*(*t*) represents the baseband jamming signal centered at *f_c_*, resulting in an in‐band jamming. The second term in Equation (6) corresponding to the jamming has a neglectable impact on the target user, owing to the low correlation between the modulation signal and the jamming across the frequency and delay domains [40]. This results in a high signal‐to‐jamming ratio (SJR), enabling accurate information recovery under jamming.

To demonstrate the proposed secure communication method, a simulation is carried out. The designed STMA comprises *N* = 40 unit cells, with the following key parameters: *f_p_* =  100 kHz, *L* = 80, *f_c_* =  23.8 GHz, *d* = 1.65 mm. The sampling frequency is set to 168 MHz, resulting in 1680 data points per symbol period *T_p_*. Additive white Gaussian noise (AWGN) is considered in the simulation with a signal‐to‐noise ratio (SNR) of 0 dB. We consider a secure communication scenario in which the intended user is located at θ_*u*_ =  0°, as shown in Figure 3a, where the +1 harmonic beam is steered toward 0°. A jammer directs interference toward the user, and an eavesdropper is placed at an arbitrary selected angle θ_*e*_ =  30°. As illustrative examples, three transmitted symbols (*s* = 1, 2, 3) with distinct frequency, delay, and phase combinations are employed to illustrate the high‐dimensional modulation; the corresponding parameters are listed in Figure 3a. The +1 harmonic beam patterns over the scanning range of [− 60°, 60°] are provided in Note S4 (Figure S5), conforming to good isolation performance. Figure 3b presents the space‐time modulation matrices U_*n*,*s*_(*t*) for symbols *s* = 1, 2, 3. Figure 3c displays the received frequency spectra across all observation angles for *s* = 1, 2, 3, respectively, normalized to the maximum value of AF^+1^(θ). The radiation power is concentrated primarily in the desired direction θ_*u*_ =  0°, while eavesdroppers at other angles receive distorted, low‐power spread‐spectrum signals, thereby maintaining low detectability. After applying the 2D correlation detection in Equation (5), Figure 3d shows the corresponding 2D correlation detection results for the intended user for symbol *s* = 1, 2, 3. Clear correlation peaks are observed, indicating the detected frequencies, delays, and phases simultaneously. A transmission of 1000 random symbols is simulated. Figure 3e,f present the statistical distribution of detected symbol frequencies and delays and the constellation diagram with an error vector magnitude (EVM) of 2.32%, respectively. The transmission performance under different levels of AWGN is presented in Note S6 (Figure S6).

To further evaluate the secure communication performance under jamming conditions, we assume an additive jamming signal is configured as an 8‐PSK waveform with the same symbol rate *f_p_* =  100 ksym/s and an SJR of 0 dB. The SNR remains 0 dB. The jamming signals dominate the frequency spectra received by the user, as shown in Figure 3g. Despite the strong interference, the 2D correlation detection results in Figure 3h still exhibit clearly distinguishable peaks, which closely match those in the jamming‐free case shown in Figure 3d. These results demonstrate accurate information recovery and strong jamming resilience. Again, a transmission of 1000 random symbols is simulated. Figure 3i,j present the statistical distribution of the detected symbol frequencies and delays and the constellation diagram with EVM = 3.92%, respectively. The robustness of the proposed scheme against other types of jamming, such as linear frequency modulation (LFM) jamming, is provided in Note S7 (Figures S7 and S8).

## 3 Results and Discussion

### 3.1 STMA Design and Measurement Setups

The STMA prototype design and measurement setups are depicted in Figure 4. The STMA comprises 40 unit cells located on two PCB layers as shown in Figure 4a. The substrates 1 and 2 are F4BM‐2 (ε_r_ = 2.2, tanδ = 0.0009) with thicknesses of 0.508 and 0.204 mm, respectively. A Rogers RO4450F prepreg (ε_r_ =  3.52, tanδ =  0.004) with a thickness of 0.2 mm is added between the substrates. An aluminum groove gap waveguide (GGW) is designed as the traveling‐wave structure. The GGW is designed to operate in the K‐band with a single TE_10_ transmission mode, ensuring slow‐wave transmission and effectively preventing radiation from the fundamental mode.

**FIGURE 4:**
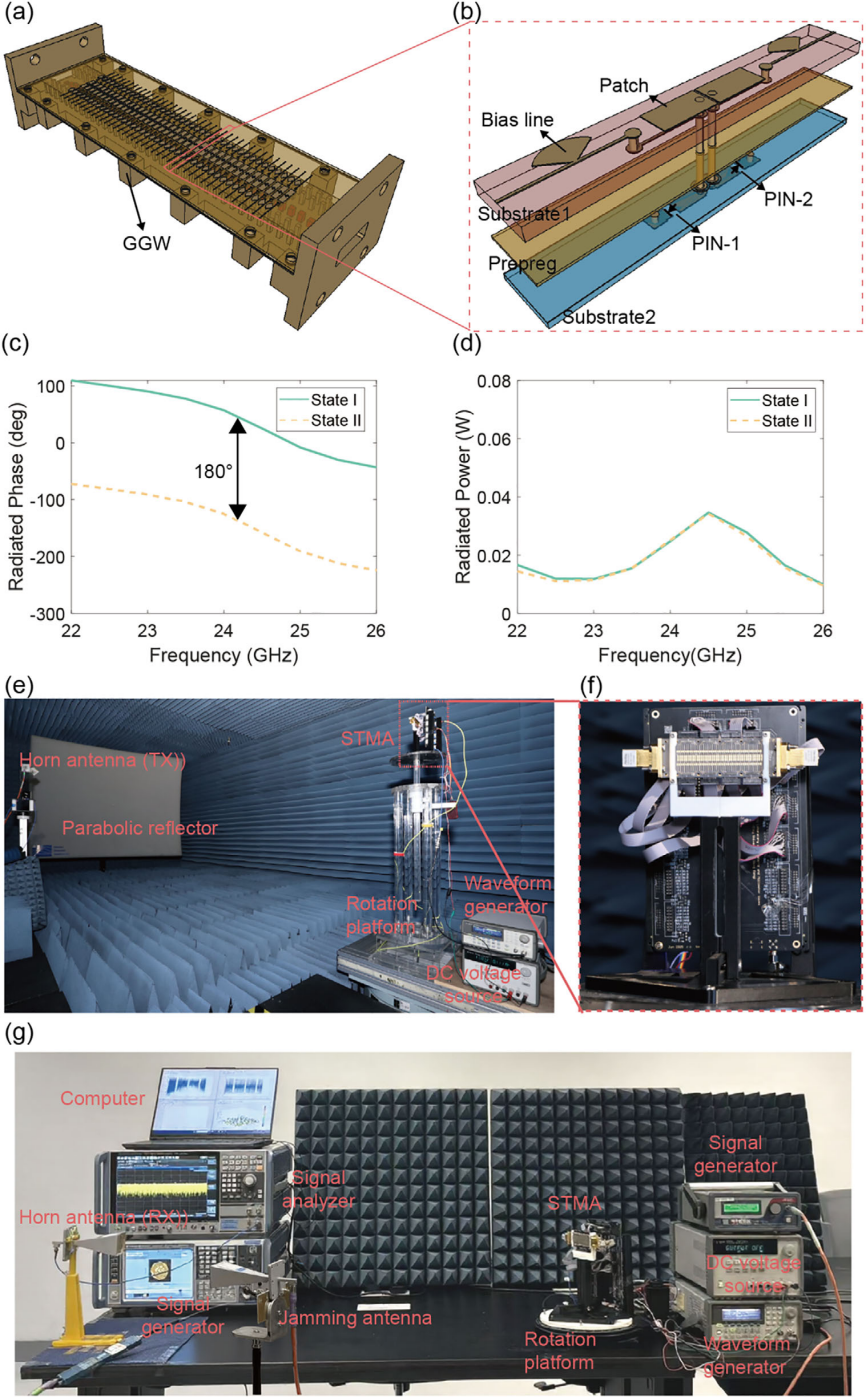
STMA configuration and experimental setups. (a) Overall structure of the STMA. (b) Configuration of the 1‐bit phase reconfigurable unit cell. Simulated unit cell responses: (c) radiated phase and (d) radiated power under two operating states. Experimental setup: (e) compact antenna test range anechoic chamber for beam pattern measurements, (f) fabricated STMA prototype, and (g) indoor environment for secure communication measurements.

The unit cell configuration is shown in Figure 4b. The spacing distance between unit cells is *d* = 1.65 mm (0.13 λ_c_ at 23.8 GHz). Each unit cell contains a pair of patches on the top layer of Substrate 1, serving as the radiating elements. The energy in the GGW is coupled to the patches via the coupling strips located on the bottom layer of Substrate 2. To enable 1‐bit phase reconfiguration, a pair of PIN diodes (MADP‐000907‐14020p) is inserted on the bottom layer of Substrate 2. Each PIN diode is controlled by a bias circuit comprising bias lines, a fan‐shaped pad, and a through‐via. The diodes’ ON/OFF states (activated at 1.33V/0V, respectively) enable 1‐bit radiation phase switching: “0” phase when PIN‐1 is ON and PIN‐2 is OFF, and “π” phase when PIN‐1 is OFF and PIN‐2 is ON. The simulated radiation performance of the unit cell in the two states under a continuous wave excitation with a power of 1 W is shown in Figure 4c,d. We observe that the radiated power remains almost unchanged, while the radiation phase difference between the two states is approximately 180°.

Far‐field beam pattern measurements are performed in a compact antenna test range anechoic chamber. The experimental setup is shown in Figure 4e. Figure 4f displays the fabricated STMA prototype with *N* = 40 unit cells. The secure communication experiment is conducted in an indoor environment, with the experimental setup illustrated in Figure 4g. The modulation signals follow the same configuration designed in Section 2, using *f_p_* =  100 kHz, *L* = 80 pulses with a pulse frequency of 8 MHz. The sampling frequency of the signal analyzer is set to 168 MHz, resulting in 1680 data points per symbol period *T_p_*.

### 3.2 Secure Communication Performance

To validate the proposed secure communication scheme against eavesdropping and jamming, a total of 1000 randomly generated symbols are transmitted. For demonstration purposes, two beam‐steering cases, pointing to 0° and −30°, are selected to evaluate the secure communication performance.

Figure 5 presents the experimental validation for a legitimate user positioned at θ_*u*_ =  0°. As shown in Figure 5a, the STMA exhibits distinct harmonic radiation characteristics, with the measured +1 harmonic forming a highly directive beam toward the intended direction. A realized gain of 11.73 dBi is achieved, while the gain difference between the desired +1 harmonic and the undesired harmonics exceeds 25 dB at 0°. The realized gain is evaluated by comparing the received power of the +1 harmonic beam with that of a standard gain horn antenna under identical measurement conditions. In the desired direction (θ_*u*_ =  0°), the received signal exhibits high power with a well‐defined spread‐spectrum profile (see inset of Figure 5a). In contrast, eavesdroppers located at θ_*e*_ =   −60°, −30°, 30°, 60° detect significantly attenuated and spectrally distorted signals. All spectra are normalized to the peak of the +1 harmonic radiation pattern to enable direct comparison. These eavesdroppers in various directions attempt to directly demodulate the received signals using the detected pulses. The resulting constellation diagrams from 1000 times demodulation attempts, shown in the insets of Figure 5a, are chaotic and indistinct. Consequently, the unauthorized observers encounter low detectability and severe signal‐space mismatch, precluding successful information recovery.

**FIGURE 5:**
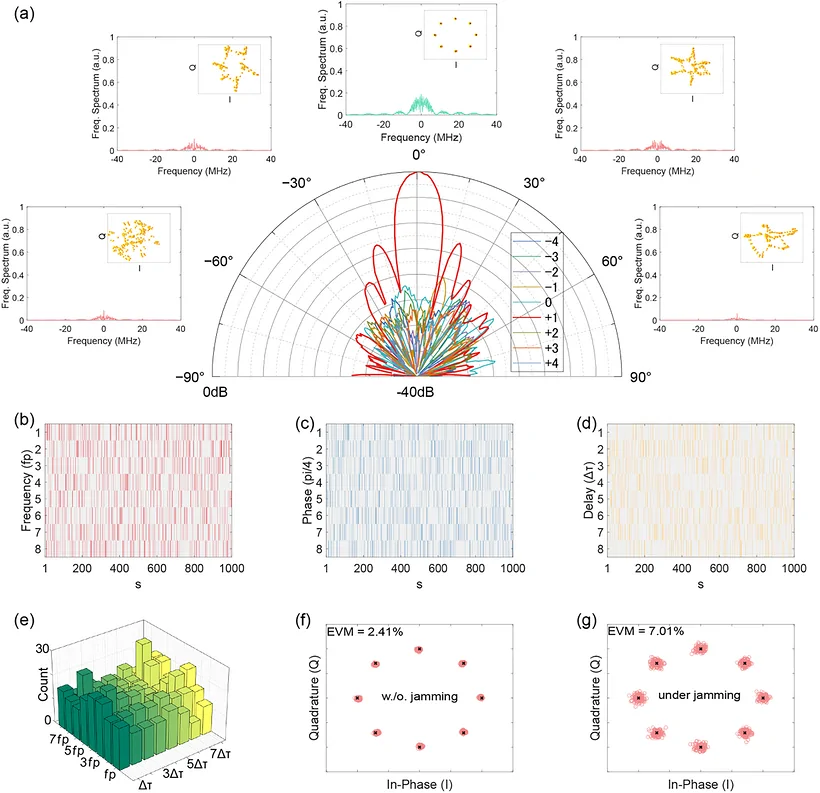
Measured secure communication performance with the user at θ_*u*_ =  0°. (a) Harmonic radiation patterns as the +1 harmonic scans to 0°, and the corresponding frequency spectra received by the user at θ_*u*_ =  0° and eavesdroppers at θ_*e*_ =   −60°, −30°, 30°, 60° (Insets: Constellation diagrams for eavesdroppers). (b)‐(d) Detected symbol frequencies, phases, and delays for 1000 transmitted symbols. (e) Statistical distribution of the detected symbol frequencies and delays. Constellation diagrams: (f) EVM = 2.41% (without jamming). (g) EVM = 7.01% (under jamming).

When the jamming antenna is inactive (see Figure 4g), the intended user correctly detects the frequencies, phases, and delays of the transmitted symbols using a nearest‐neighbor rule, as shown in Figure 5b–d. Figure 5e,f further present the statistical distribution of the detected symbol frequencies and delays, together with the constellation diagram, which exhibits an EVM of 2.41%, confirming accurate information recovery in the desired direction. Detailed time‐domain waveforms and corresponding 2D correlation detection results for representative measured symbols are provided in Note S8 (Figure S9).

When the jamming antenna is activated (see Figure 4g), the jamming signal is an 8‐PSK waveform with a symbol rate of *f_p_* =  100 ksym/s, and 1000 randomly generated 8‐PSK symbols are transmitted from the jammer. The input power of the jamming antenna is adjusted to maintain an SJR of approximately 0 dB. The detected frequencies, phases, and delays for the 1000 transmitted symbols, obtained via the same nearest‐neighbor rule, are identical to those in the jamming‐free case (Figure 5b–d). Moreover, the statistical distribution of symbol frequencies and delays remains the same as that without jamming, as shown in Figure 5e. Figure 5g displays the corresponding demodulated constellation diagram, which exhibits an EVM of 7.01%. The measured constellation shows increased dispersion around the ideal positions compared with the jamming‐free case in Figure 5f, due to distortion introduced by the jamming signal. Nevertheless, the measured EVM remains low, and the constellation points remain clearly distinguishable, demonstrating the strong anti‐jamming capability of the proposed method.

Figure 6 presents the measured results for the security communication when the user is located at θ_*u*_ =   −30°. Figure 6a presents the measured radiation patterns of the STMA at different harmonic frequencies, demonstrating a realized gain of 9.88 dBi for the desired +1 harmonic. At −30°, the gain difference between the desired +1 harmonic and other harmonics exceeds 23 dB. The received spectrum in the desired direction θ_*u*_ =   −30° exhibits high power with a distinct spread‐spectrum profile, whereas the spectra received by the eavesdroppers positioned at θ_*e*_ =   −60°, 0°, 30°, 60° are weak, distorted, and highly spread. All spectra are normalized to the maximum value of the +1 harmonic beam pattern. Again, these eavesdroppers attempt to directly demodulate the received signals using the detected pulses. The resulting constellation diagrams from 1000 times demodulation attempts, illustrated in the insets of Figure 6a, are chaotic and indistinct. Consequently, the eavesdroppers experience low detectability and cannot recover the transmitted information due to severe signal‐space dimensional mismatch.

**FIGURE 6:**
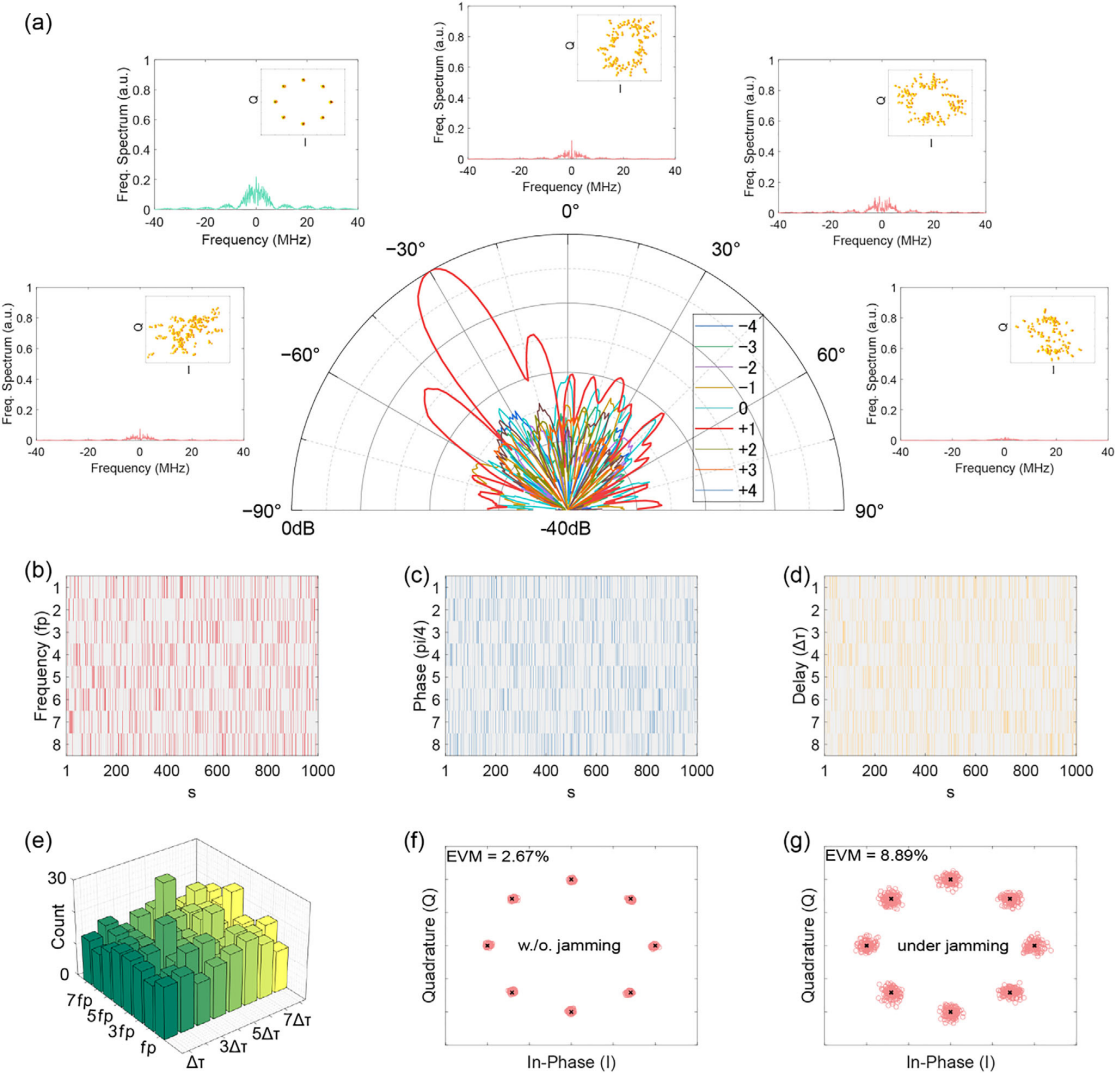
Measured secure communication performance with the user at θ_*u*_ =   −30°. (a) Harmonic radiation patterns as the +1 harmonic scans to −30°, and the corresponding frequency spectra received by the user at θ_*u*_ =   −30° and eavesdroppers at θ_*e*_ =   − 60°, 0°, 30°, 60° (Insets: Constellation diagrams for eavesdroppers). (b–d) Detected symbol frequencies, phases, and delays for 1000 transmitted symbols. (e) Statistical distribution of the detected symbol frequencies and delays. Constellation diagrams: (f) EVM = 2.67% (without jamming). (g) EVM = 8.89% (under jamming).

When the jamming antenna is inactive, the intended user correctly detects the frequencies, phases, and delays of the transmitted symbols, as shown in Figure 6b–d. The statistical distributions of the detected symbol frequencies and delays and the constellation diagram are shown in Figure 6e,f, yielding an EVM of 2.67% and confirming accurate information recovery in the desired direction. Representative measured time‐domain waveforms and their corresponding 2D correlation detection results are provided in Note S8 (Figure S10).

When the jamming antenna is activated, an 8‐PSK jamming signal with a symbol rate of *f_p_* =  100 ksym/s is transmitted, and the input power is adjusted to maintain an SJR of approximately 0 dB. Using the same detection rule, the recovered frequencies, phases, and delays remain consistent with the jamming‐free case (Figure 6b–d), and the statistical distributions of symbol frequencies and delays are unchanged (Figure 6e). The constellation in Figure 6g exhibits an EVM of 8.89%. Although increased dispersion appears due to jamming‐induced distortion, the constellation points remain clearly distinguishable, demonstrating the strong anti‐jamming capability of the proposed STMA.

According to 3GPP TS 45.005 (www.3gpp.org), the RMS EVM requirement for 8‐PSK modulation is specified as 9.0% under normal conditions and 10.0% under extreme conditions for Mobile Stations (MS). For Base Transceiver Stations (BTS), the EVM limits range from 7.0% to 9.0%, depending on the measurement scenario. In the proposed system, the measured EVM values under jamming‐free conditions are 2.41% and 2.67%, which are substantially lower than the EVM limits specified by the 3GPP standard. Even under jamming conditions, the measured EVM values remain at 7.01% and 8.89%, still satisfying the EVM requirements for MS and BTS operations. These results confirm that the proposed system achieves communication performance compatible with practical telecommunication standards while maintaining robustness against interference.

The high‐dimensional modulation implemented by the STMA inherently integrates spatial directivity with spread‐spectrum‐based LPD, while enabling agile frequency and delay varying across symbols. Rather than relying solely on power dispersion, the security enhancement in this design arises from the multidimensional structure of the EM mapping, which substantially complicates detection and parameter estimation by unauthorized observers. Moreover, the modulation exhibits intrinsic resilience to in‐band jamming. Owing to the weak correlation between the multidimensional modulation states and interfering signals across the frequency and delay domains, coherent energy accumulation by the jammer becomes ineffective.

## 4 Conclusion

In this paper, we introduce a high‐dimensional EM modulation paradigm for physical layer security enabled by an STMA. By employing direct modulation of the +1 harmonic with independently programmable phase and frequency, as well as pseudorandom modulation with adjustable time‐delay, information is embedded across multiple orthogonal or weakly correlated EM degrees of freedom. This multidimensional mapping intrinsically embeds information into the physical radiation process, transforming the STMA into an active information‐processing platform. Security and spectral efficiency enhancements arise from the expansion of the effective EM state space rather than from relying only on conventional bandwidth inflation or power redistribution. The resulting transmission simultaneously exhibits spatial directivity, agile frequency‐delay varying, LPD, and strong resilience to both jamming and eavesdropping. Note that all these functionalities are consolidated within a compact waveguide‐integrated STMA employing PIN‐diode‐based 1‐bit reconfigurable phase states, yielding a hardware‐efficient and scalable architecture. A comprehensive comparison between the proposed method and state‐of‐the‐art space‐time modulation approaches for wireless communications is provided in Note S9 (Table S3).

## 5 Methods

The theoretical framework formulated in Equations (1)–(6) is numerically implemented using MATLAB. The design of the STMA and 1‐bit programmable unit cell (as shown in Figure 4a,b) is performed through full‐wave numerical electromagnetic simulations conducted in CST Microwave Studio 2019 (https://www.3ds.com/products/simulia/cst‐studio‐suite).

As shown in Figure 4e, the beam pattern measurement is conducted in a compact antenna test range anechoic chamber. A Rohde & Schwarz SMW200A signal generator feeds a standard gain horn to produce a 23.8 GHz CW signal. The transmitting horn's spherical wave is converted to a plane wave by a parabolic reflector. The STMA prototype, comprising 40 unit cells as designed (see Figure 4f), is mounted on a rotation platform within the reflector's quiet zone. The STMA's output connects to a Rohde & Schwarz FSW43 signal analyzer (2Hz–43.5GHz), while a waveform generator and DC voltage source provide clock signals and power for the FPGA controller. All system components – including the signal generator, analyzer, rotation platform motor, and FPGA board – are synchronized to ensure measurement stability.

As shown in Figure 4g, the secure communication measurement is conducted in an indoor environment. A signal generator excites the STMA with a 23.8 GHz CW signal with an input power of 20 dBm, while a waveform generator and DC power supply provide the clock and bias voltages for the FPGA controller. The STMA prototype, consisting of 40 unit cells as designed, is mounted on a rotation platform. The receiving (RX) antenna is connected to a Rohde & Schwarz FSW43 signal analyzer (2 Hz–43.5 GHz). A jamming antenna, driven by a Rohde & Schwarz SMW200A signal generator, transmits an 8‐PSK waveform directly to the RX antenna. The STMA is positioned 1.3 m away from the RX antenna, ensuring that the STMA operates within the far‐field region. All system components, including the signal generator, signal analyzer, rotation motor, and FPGA board, are synchronized to ensure stable measurements.

## Author Contributions

**Zheng Xing Wang**: methodology, validation. **Chenfeng Yang**: methodology, validation. **Geng‐Bo Wu**: conceptualization, methodology, validation, investigation, writing – review and editing, supervision. **Xinyu Fang**: conceptualization, investigation, methodology, validation. **Yiqing Sun**: conceptualization, investigation, methodology, validation, writing – original draft. **Siran Wang**: validation, investigation. **Kaixu Wang**: writing – review and editing, methodology.

## Conflicts of Interest

The authors declare no conflicts of interest.

## Supporting information




**Supporting File**: advs76676‐sup‐0001‐SuppMat.docx.

## Data Availability

The data that support the findings of this study are available from the corresponding author upon reasonable request.
