# Supplementary material for: Space‐Time Metasurface Antenna Enabled High‐Dimensional Electromagnetic Modulation for Intrinsic Physical Layer Security
Source: Adv Sci (Weinh). 2026 Jul 20:e76676. Online ahead of print. doi: 10.1002/advs.76676 (PMC13383151; doi:10.1002/advs.76676)
Supplement: Supplementary file 1 — Supporting File: advs76676‐sup‐0001‐SuppMat.docx. [file ADVS-9999-e76676-s001.docx]

Supporting Information

Space-Time Metasurface Antenna Enabled High-Dimensional Electromagnetic Modulation for Intrinsic Physical Layer Security

Xinyu Fang, Yiqing Sun, Chenfeng Yang, Siran Wang*, Zhengxing Wang*, Kaixu Wang and Geng-Bo Wu*

Xinyu Fang, Yiqing Sun, Chenfeng Yang, Siran Wang, Zhengxing Wang, and Geng-Bo Wu

State Key Laboratory of Terahertz and Millimeter Waves and Department of Electrical Engineering

City University of Hong Kong, Hong Kong, SAR 999077, China

E-mail: bogwu2@cityu.edu.hk.

Kaixu Wang

School of Electronics and Information Engineering, Harbin Institute of Technology, Shenzhen, China

**Supplementary Note 1. Direct modulation of the +1 harmonic for QAM**

For QAM, both the amplitude and phase of the transmitted symbols must be controlled. In the proposed framework, QAM can be realized by combining optimization-based amplitude modulation, such as implemented using a genetic algorithm (GA), with time-delay-induced phase modulation.

As an illustrative example, a 16-QAM scheme for the intended user at 0° is presented. As shown in Fig. S1(a), the achieved 16-QAM constellation consists of three normalized-amplitude rings with amplitudes of 1 (red outer ring), $\sqrt{5}$/3 (green middle ring), and 1/3 (yellow inner ring). The corresponding +1 harmonic beam patterns are shown in Fig. S1(b). The +1 harmonic beam pattern of the outer ring is generated using the modulation matrix shown in Fig. S1(c) (Fig. 2(b) in the main text), denoted as $\mathrm{AF}_{\mathrm{outer}}^{+1}(\theta)$. Taking this beam pattern as the reference, the modulation matrices for the middle and inner rings are obtained through GA-based optimization with target amplitudes defined as $\mathrm{AF}_{\mathrm{Middle}}^{+1}(0^{\circ})=\sqrt{5}\mathrm{AF}_{\mathrm{outer}}^{+1}(0^{\circ})/3$ and $\mathrm{AF}_{\mathrm{Inner}}^{+1}(0^{\circ})=\mathrm{AF}_{\mathrm{outer}}^{+1}(0^{\circ})/3$.

As a result, the optimization process ensures the desired amplitude ratios among the three rings while maintaining a common initial phase for the corresponding beam patterns at 0°. Subsequently, controllable phase states are realized by introducing appropriate time delays into the direct modulation of the +1 harmonic. The outer-ring constellation points are generated using the modulation matrices shown in Figs. S1(c)-(f), whereas the middle- and inner-ring constellation points are obtained using the optimized modulation matrices shown in Figs. S1(g)-(n) and Figs. S1(o)-(r), respectively. Consequently, simultaneous amplitude and phase control of the +1 harmonic is achieved, enabling the realization of 16-QAM modulation.


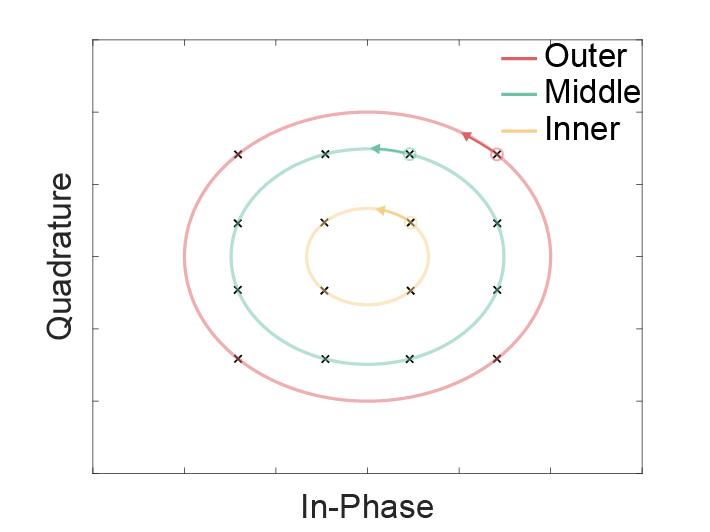

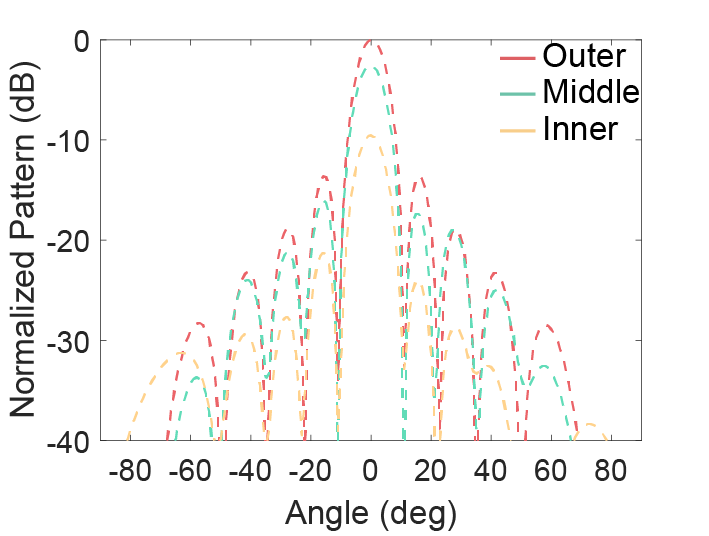


(a) (b)

(c) (d) (e) (f)

(g) (h) (i) (j)

(k) (l) (m) (n)

(o) (p) (q) (r)

**Figure S1.** Modulation scheme for 16-QAM. (a) Achieved 16-QAM constellation diagram with three normalized-amplitude rings: 1 for the red outer ring, $\sqrt{5}$/3 for the green middle ring, and 1/3 for the yellow inner ring. (b) Corresponding +1 harmonic beam patterns for the user at 0°. (c)-(f), (g)-(n), and (o)-(r) Direct modulation of the +1 harmonic with controllable phases along the outer, middle, and inner rings, respectively.

Fig. S2 presents the simulated 2-D correlation detection results for the proposed 16-QAM scheme, with all results normalized to the maximum value in Fig. S2(a). To focus on the demonstration of amplitude and phase modulation, the frequency and delay states are arbitrarily selected and kept unchanged for all symbols, with $f_{s}=f_{p}$, $\tau_{s}=5\Delta\tau$. Figs. S2(a)-(d), (e)-(l), and (m)-(p) correspond to the outer, middle, and inner constellation rings, respectively. Well-defined correlation peaks are observed for all constellation points, demonstrating the accurate detection and recovery of the frequency, delay, and phase information required for 16-QAM demodulation.

(a) (b) (c) (d)

(e) (f) (g) (h)

(i) (j) (k) (l)

(m) (n) (o) (p)

**Figure S2**. Simulated 2D correlation detection results for 16-QAM. (a)-(d), (e)-(l), and (m)-(p) show the results for the outer, middle, and inner rings, respectively.

**Supplementary Note 2. Autocorrelation of the Pseudorandom Sequence *C*(*t*)**

Fig. S3(a) illustrates the pseudorandom modulation waveform employed in this work with *L* = 80, while Fig. S3(b) presents its corresponding autocorrelation function. A prominent main peak is clearly observed, whereas the highest sidelobe remains at an amplitude level of approximately 0.2, demonstrating favorable correlation characteristics. As the time delay τ*_s_* changes, the main autocorrelation peak shifts accordingly, which accurately indicates the corresponding delay position.

(a) (b)

**Figure S3.** (a) Time-domain waveform of the pseudorandom modulation signal *c*(*t*). (b) Corresponding autocorrelation performance.

**Supplementary Note 3. Undesired Harmonics Suppression Mechanism**

In the proposed system, the +1 harmonic is intentionally selected as the desired frequency component for radiation and communication, while radiation at the fundamental frequency and other undesired harmonics is significantly suppressed, thereby enabling sideband-free operation. This selective harmonic radiation originates from the dispersion characteristics of the guided wave, which allow only the +1 harmonic to satisfy the radiation condition. To clarify this mechanism, the corresponding dispersion diagram has been added in Fig. S4.

**Figure S4.** Dispersion diagram for frequency conversion and harmonic suppression.

The fundamental guided mode supported by the groove gap waveguide is represented by the blue curve. At the carrier frequency *f_c_*, the guided wave operates in the slow-wave region outside the light cone and therefore does not satisfy the radiation condition. The corresponding phase constant, extracted from full-wave simulations of the STMA loaded with PIN diodes, is $\xi_{wg}={1.15\xi}_{c}$.

When space-time modulation is introduced, a set of harmonic frequencies is generated at $f_{c}+hf_{s}$, where $h=\pm1, \pm2\ldots$ denotes the harmonic order. As shown in Fig. S4, the yellow, red, and green curves correspond to the -1, +1, and +2 harmonics, respectively. By applying a properly designed space-time modulation matrix with a phase-delay gradient, $\alpha_{n}^{+1}=e^{-j\xi_{c}(n-1)d\sin\theta_{u}}e^{j\xi_{wg}(n-1)d}$, the desired +1 harmonic is selectively shifted into the fast-wave region (red dot in Fig. S4), thereby satisfying the radiation condition and enabling efficient radiation. In contrast, the -1 and +2 harmonics (yellow and green dots in Fig. S4), together with higher-order harmonics, remain outside the light cone in the slow-wave region and are therefore non-radiative, similar to the fundamental mode. Furthermore, these undesired harmonics experience substantial phase mismatch in the groove gap waveguide, resulting in additional suppression during propagation. Consequently, the proposed STMA exhibits an inherent self-filtering effect that selectively radiates the desired harmonic while suppressing unwanted harmonic components [25].

**Supplementary Note 4. The +1 Harmonic Beam Steering Performance**

The *n*^th^ unit cell signal $\text{m}_{n}(t)$ is a time-delayed version of $\text{m}(t)$, *i.e.*, $\text{m}_{n}(t)=m(t-\mu_{n})$, $\mu_{n}\in[0,T_{s}]$. According to Fourier properties, the *h*^th^ harmonic coefficient is given by $\alpha_{n}^{h}={Ae}^{-j2\text{π}{hf}_{s}\mu_{n}}$, where *A* is a constant. By appropriately designing the time-delay at different unit cell as

$$\mu_{n}=\frac{\xi_{c}(n-1)d\sin\theta_{u}{-\xi}_{wg}(n-1)d}{2\text{π}f_{s}}$$

(S1)

The +1 harmonic can be steered toward the desired direction $\theta_{u}$, *i.e.*, momentum manipulation, as shown in Fig. 2(d) of the main text. In addition, the modulation signal of each unit cell $\text{m}_{n}(t)$ can be independently optimized to tailor harmonic radiation characteristics, such as the peak gain value, sidelobe level, and sideband level, etc.

Fig. S5 illustrates the simulated beam-steering radiation performance of the +1 harmonic. In Fig. S5(a), the +1 harmonic radiation pattern is scanned over the range of [-60°, 60°] by setting the the delay $\mu_{n}$ of $\text{m}_{n}(t)$ according to (S1). All patterns are normalized to the maximum value for the +1 harmonic radiation pattern scanning to 0°. The maximum scanning loss is approximately 6 dB over the scanning range of [-60°, 60°]. Fig. S5(b) shows the isolation, defined as the gain difference between the +1 harmonic and other undesired harmonic components at the steering angle, exceeds 25 dB for all the scanning cases, showing favorable single side band scanning performance.

(a) (b)

**Figure S5.** Simulated +1 harmonic beam steering performance. (a) Radiation patterns over the scanning range of [-60°, 60°]. (b) Isolation between the +1 harmonic and others harmonic components.

**Supplementary Note 5. Spectral Efficiency Calculation**

Spectral efficiency, also referred to as bandwidth efficiency, is a core performance metric in wireless and wired communication systems. It quantifies the amount of data transmitted per unit of bandwidth over a given time, reflecting how efficiently a communication system utilizes the limited radio frequency spectrum resource. Spectral efficiency is defined as the ratio of the achievable data rate (or throughput) to the occupied bandwidth of the communication system. Its standard unit is bits per second per hertz (bit/s/Hz) (or bps/Hz). The spectral efficiency is expressed as

$$\eta=R_{d}/B_{w}$$

(S2)

where $R_{d}$ is effective data rate (bit/s), *i.e.*, the actual number of bits successfully transmitted per second (excluding overhead such as error correction codes, framing, or pilot signals). $B_{w}$ is occupied bandwidth (Hz), *i.e.*, the bandwidth allocated for signal transmission (e.g., channel bandwidth in cellular systems, or the 3 dB bandwidth of the transmitted signal).

In this work, the data rate is given by ${{(log}_{2}KMQ)}/{T_{p}}$ bit/s. Since the variation range of the +1 harmonic modulation frequency $f_{s}=kf_{p}$, $k\in\left\{ 1,...,8 \right\}$, is much smaller than the spread-spectrum bandwidth $Lf_{p}$, *L* = 80, the occupied bandwidth can be approximated as $Lf_{p}$. Therefore, the spectral efficiency of the proposed method is expressed as $\eta=\frac{{log}_{2}KMQ}{L}$ bit/s/Hz.

**Supplementary Note 6. Transmission Performance under Noise**

To further evaluate the secure communication performance, we simulate the transmission of 1000 random symbols with additive white Gaussian noise (AWGN) at specified SNR levels. As an illustrative example, the space-time modulation matrix $U_{n,s}(t)$ for the user located at 0° and symbol *s* = 1 is employed. Fig. S6 shows the resulting error vector magnitude (EVM) as a function of SNR. The SNR is varied from -15 dB to 20 dB in 5 dB increments. As expected, the EVM increases with decreasing SNR, yet remains below 10 % for SNRs above -11.3 dB, demonstrating the robustness of the proposed method.

**Figure S6.** EVM performance as a function of SNR.

**Supplementary Note 7. Secure Transmission Performance against LFM Jamming**

To further demonstrate the robustness of the proposed method against jamming, a linear frequency modulation (LFM) jamming signal is considered, with a period of $T_{p}=10 \mu s$, a bandwidth of *B* = 1 MHz, and SJR of 0 dB, as shown in Fig. S7. The SNR remains 0 dB. Although the jamming signal dominates the frequency spectrum received by the user for symbol *s* = 1 (Fig. S8(a)), the 2-D correlation detection result in Fig. S8(b) still exhibits a clearly distinguishable peak that matches the one obtained in the jamming-free case (Fig. 3(d) in the main text). These results confirm accurate information recovery and strong resilience to jamming.

A transmission of 1000 random symbols is simulated. Figs. S8(c) and (d) present the statistical distribution of the detected symbol frequencies and delays, and the constellation diagram with an EVM of 5.32 %, respectively. The characteristics of the pseudorandom sequence, including the number of sub-pulses *L* and the distribution of binary code $\{1,-1\}$, can be optimized [45] to further suppress the sidelobes shown in Fig. S8(b).

**Figure S7.** LFM jamming waveform in the (a) time domain and the (b) frequency domain.

(a) (b)

(c) (d)

**Figure S8.** Simulated secure communication performance against LFM jamming. (a) Frequency spectrum under LFM jamming of the user at $\theta_{u}=0^{\circ}$ for symbol *s* = 1. (b) 2-D correlation results detected by the user for symbol *s* = 1. 1000 random symbols transmission: (c) Statistical distribution of detected symbol frequencies and delays. (d) Constellation diagram with EVM = 5.32 %.

**Supplementary Note 8. Time-Domain Waveforms and Correlation Results of Measured Symbols**

To provide representative examples of the proposed high-dimensional modulation scheme, the first three transmitted symbols *s* = 1, 2, 3, each characterized by a unique combination of frequency, delay, and phase states, are selected for detailed analysis. These symbols correspond to the measured results shown in Figs. 5 and 6 of the main text for the intended users located at 0° and −30°, respectively. The associated modulation parameters are listed in Tables S1 and S2.

Figs. S9 and S10 show the measured time-domain waveforms together with their corresponding 2-D correlation detection results. Well-defined correlation peaks are observed for all three symbols, demonstrating the accurate recovery of the frequency, delay, and phase information.

**Table S1.** Parameters of the transmitted symbols for *s* = 1, 2, 3 in Fig. 5.

| EM dimension | s = 1 | s = 2 | s = 3 |
| --- | --- | --- | --- |
| Frequency | 7 | 1 | 2 |
| Phase | 7 | 1 | 7 |
| Delay | 5 | 4 | 7 |

(a) (b) (c)

(d) (e) (f)

(g) (h) (i)

**Figure S9.** Measured transmitted symbols corresponding to Fig. 5. (a), (d), (g) Time-domain amplitude waveforms; (b), (e), (h) time-domain phase waveforms; and (c), (f), (i) 2-D correlation detection results for symbols *s* = 1, 2, 3, respectively.

**Table S2.** Parameters of the transmitted symbols for *s* = 1, 2, 3 in Fig. 6.

| EM dimension | s = 1 | s = 2 | s = 3 |
| --- | --- | --- | --- |
| Frequency | 6 | 6 | 8 |
| Phase | 4 | 1 | 6 |
| Delay | 1 | 3 | 2 |

(a) (b) (c)

(d) (e) (f)

(g) (h) (i)

**Figure S10.** Measured transmitted symbols corresponding to Fig. 6. (a), (d), (g) Time-domain amplitude waveforms; (b), (e), (h) time-domain phase waveforms; and (c), (f), (i) 2-D correlation detection results for symbols *s* = 1, 2, 3, respectively.

**Supplementary Note 9. Comparison between the Proposed Method and State-of-the-Art Methods**

Table S3 compares the proposed method with state-of-the-art space-time modulation approaches for physical-layer security communicaiton reported in the literature. By jointly exploiting +1 harmonic direction modulation with independently programmable phase and frequency, together with pseudorandom modulation incorporating programmable time-delay, this work directly embeds information across three EM dimensions and transmitted securely. The resulting signaling mechanism simultaneously achieves spatially directional transmission, agile frequency-delay varying, and LPD. This expansion of the EM state space provides enhanced spectral efficiency and resilience against both jamming and eavesdropping. All functionalities are realized within a compact waveguide-integrated STMA using only 1-bit reconfigurable phase states, resulting in a hardware-efficient architecture.

**Table S3.**  Comparison between the Proposed Method and State-of-the-Art Methods

| Method | Platform | Information mapping | Security | Spectral efficiency (bit/s/Hz)^c)^ | Sideband free | Direct information modulation | Jamming protection | LPD |
| --- | --- | --- | --- | --- | --- | --- | --- | --- |
| [28] | 1-bit STMA^a)^ | 1D: Phase;  8PSK | Directional modulation | ${log}_{2}M$ | Yes | Yes | No | No |
| [29] | 2-bit STMM^b)^ | 1D: Frequency;  BFSK | No | $\frac{{log}_{2}K}{T_{p}(\Delta f+{1/T}_{p})}$ | No | Yes | No | No |
| [30] | 1-bit STMM | 1D: Phase;  QPSK | Directional modulation | ${log}_{2}M$ | No | No | No | No |
| [31] | 1-bit STMM | 1D: Phase;  BPSK | Pseudorandom modulation | $\frac{{log}_{2}M}{L}$ | No | No | Yes | Yes |
| [32] | 1-bit STMM | 1D: Phase;  QPSK | No | ${log}_{2}M$ | No | Yes | No | No |
| [33] | 2-bit STMM | 1D: Frequency;  BFSK | Directional modulation | $\frac{{log}_{2}K}{T_{p}(\Delta f+{1/T}_{p})}$ | No | Yes | No | No |
| [34] | 1-bit STMM | 1D: Phase;  BPSK | Pseudorandom modulation | $\frac{{log}_{2}M}{L}$ | No | Yes | Yes | Yes |
| [35] | 1-bit STMM | 1D: Code;  4 code states | Pseudorandom modulation | $\frac{{log}_{2}Q}{L}$ | No | Yes | Yes | Yes |
| [36] | 1-bit STMM | 1D: Phase;  QPSK | No | ${log}_{2}M$ | No | Yes | No | No |
| This work | 1-bit STMA | 3D: Frequency, phase, delay;  8 frequencies, 8 Phases, 8 delays | EM state space expansion + Pseudorandom modulation | $\frac{{log}_{2}KMQ}{L}$ | Yes | Yes | Yes | Yes |

1. STMA, space-time metasurface antenna; ^b)^ STMM, space-time modulated metasurface. ^c)^ *K*, *M*, *Q* are the numbers of frequency, phase, and code states, respectively; *L* is the number of pseudorandom sub-pulses. $T_{p}$ is the symbol period. $\Delta f$ is the frequency spacing between two FSK symbols.
